# Supplementary material for: k-mer manifold approximation and projection for visualizing DNA sequences
Source: Genome Res. 2025 May;35(5):1234–46. doi: 10.1101/gr.279458.124 (PMC12047656; doi:10.1101/gr.279458.124)
Supplement: Supplement 8 [file Supplemental_Data_1.zip › Supplemental_Data_1/XBP1_TGTTCA20NGA_AC_3/XBP1_TGTTCA20NGA_AC_3_PCA.pdf]

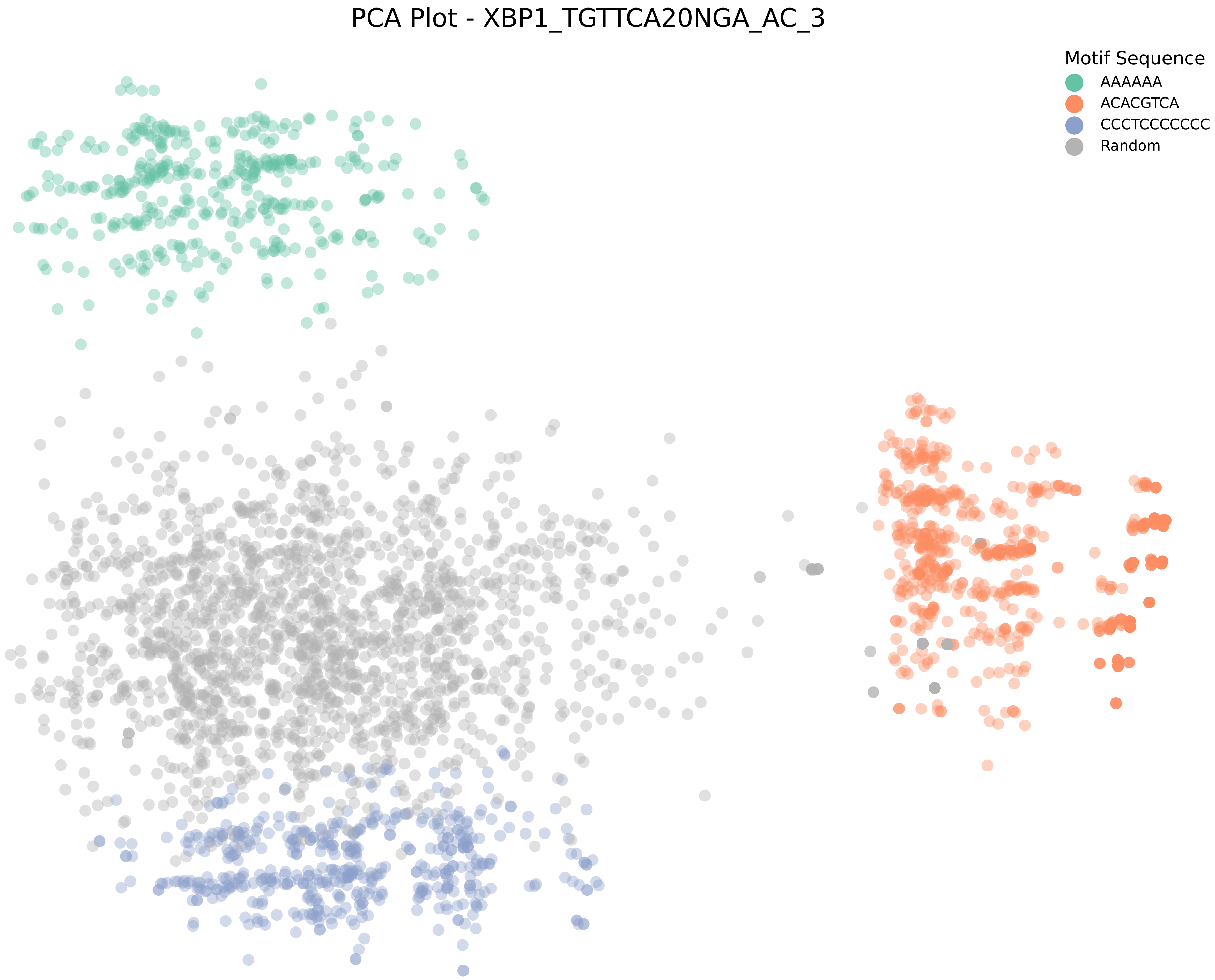
PCA Plot - XBP1_TGTTCA20NGA_AC_3
Motif Sequence
AAAAAA
ACACGTCA
CCCTCCCCCCC
Random
